# A Novel Point Cloud Registration Method Based on ROPNet

**DOI:** 10.3390/s23020993

**Published:** 2023-01-15

**Authors:** Yuan Li, Fang Yang, Wanning Zheng

**Affiliations:** The Engineering Research Center of Metallurgical Automation and Measurement Technology, Wuhan University of Science and Technology, Wuhan 430081, China

**Keywords:** deep learning, point cloud registration, cross-entropy loss, channel attention mechanism

## Abstract

Point cloud registration is a crucial preprocessing step for point cloud data analysis and applications. Nowadays, many deep-learning-based methods have been proposed to improve the registration quality. These methods always use the sum of two cross-entropy as a loss function to train the model, which may lead to mismatching in overlapping regions. In this paper, we designed a new loss function based on the cross-entropy and applied it to the ROPNet point cloud registration model. Meanwhile, we improved the ROPNet by adding the channel attention mechanism to make the network focus on both global and local important information, thus improving the registration performance and reducing the point cloud registration error. We tested our method on ModelNet40 dataset, and the experimental results demonstrate the effectiveness of our proposed method.

## 1. Introduction

Point cloud registration is the basis of 3D reconstruction [1,2], 3D localization [3,4,5,6], pose estimation [7,8,9], and other fields. With the development of deep learning, point cloud registration develops from traditional iterative closest point-based (ICP) [10] methods to approaches based on deep learning, such as PCRNet [11], D3feat [12], iterative distance-aware similarity matrix convolution network (IDAM) [13], point cloud registration with deep attention to the overlap region (PREDATOR) [14], robust point matching (RPMNet) [15], etc. In recent years, these methods have achieved good registration results under certain conditions. However, most of the current models do not perform well in overlapping regions. In this paper, to solve the above issue, we will take measures from two aspects, i.e., the loss function and the backbone network. To demonstrate the effectiveness, we use the representative overlapping points network (ROPNet) [16] as an example in the following context.

Loss functions measure the difference between the real value and the predicted value in deep learning and play important roles in back-propagation. Depending on different tasks, loss functions can be roughly divided into two categories. The first one is used for regression tasks, such as mean absolute (MAE), mean square error (MSE), Huber loss, etc. The other one is used for classification tasks, such as focal loss [16], center loss [17], circle loss [18], dice loss [19], cross-entropy loss, etc. The point cloud registration task involves the classification problem, and often uses the cross-entropy as the loss function. However, researchers [19,20] found that, in some cases cross-entropy loss can not achieve good results. For example, when the data between positive and negative samples are seriously unbalanced, negative samples will dominate the training of the model. In this case, by using the original cross-entropy loss, the model cannot predict accurately. To solve this problem, many researchers have made a lot of improvements based on the original cross-entropy, such as weighted cross-entropy (WCE) [21], balanced cross-entropy (BCE) [22], dice loss [19], focal loss [20], etc. Most of the above approaches improve the cross-entropy loss by introducing weights; however, these metrics often neglect the relationship between the corresponding points in overlapping regions.

In this paper, to improve the registration quality on overlapping areas, we introduce a new way of calculating loss function based on the cross-entropy. Specifically, we use the product of two cross-entropy loss instead of the sum. We prove the validity of our proposed loss function in theory in Section 3.1. Moreover, to testify the effectiveness of the proposed loss, we apply it to a recently proposed network, i.e., the ROPNet, which originally uses the sum of two cross-entropy to supervise the overlapping area. In addition, the ROPNet considers more global information but ignores local information, which leads to mis-registration at places where there are details. Therefore, we propose to add a channel attention mechanism to focus on important local information, and use it to complement the self-attention mechanism in the original model. A large number of experiments show that, compared with the original ROPNet scheme, the proposed method has a certain improvement in overlapping point cloud registration. Our main contributions are as follows:

• A new loss function based on cross-entropy is designed, which helps improve the point clouds registration accuracy, especially in overlapping regions.
• The performance of the ROPNet is also improved by adding a channel attention mechanism.
• A large number of experiments on commonly used datasets were conducted and verified the effectiveness of our proposed method.

The rest of the paper is organized as follows. Section 2 introduces the related work. In Section 3, a new loss function based on the cross-entropy loss is proposed. Experiments are presented in Section 4 and conclusions are drawn in Section 5.

## 2. Related Work

In this section, we introduce some related work and preliminary knowledge, including the ROPNet model, cross-entropy loss, and attention mechanism.

### 2.1. ROPNet

**Figure 1:**
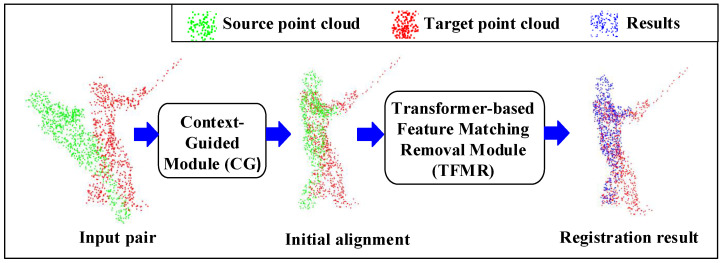
The original point cloud registration model of ROPNet.

ROPNet is a point cloud registration model that typically uses representative points in overlapping regions for registration. As shown in Figure 1, the ROPNet consists of a context-guided (CG) module and a transformer-based feature matching removal (TFMR) module.

Generally speaking, ROPNet can be divided into two stages: coarse alignment and fine alignment. In the first stage, the source point cloud and the target point cloud are input. The CG module is used to extract global features from the input and compute the overlap score between two point clouds. The extracted global features are then used for coarse alignment, and the non-representative points are removed based on the overlap score. In the second stage, point features are generated based on the offset attention [23,24]. The representative points are used to generate accurate correspondences. Finally, the precise registration of point clouds is carried out.

### 2.2. Cross-Entropy Loss

The cross-entropy loss is a widely used loss function to classification problems. In the point cloud registration model [16,25], *p* is defined as the true distribution. It can be written as

(1) $$p\left(x_{i}\right)=\left\{\begin{array}{cc} 1 & if\;i\;=\;j\\ 0 & \mathrm{otherwise} \end{array}\right.,$$

where $x_{i}$ refers to any point in the source point cloud. If the point in the source point cloud matches the point in the target point cloud, which means i=j, then $p(x_{i})=1$. Otherwise, $p(x_{i})=0$.

At the same time, *q* is defined as the predicted probability distribution, which represents the probability that the source point cloud matches the target point cloud. It can be written as:

(2) $$q\left(x_{i}\right)=\frac{e^{{}_{{}_{x_{i}}}}}{\sum_{j=1}^{n}e^{{}_{{}_{x_{j}}}}},$$

where *n* represents the number of points in the point cloud.

Then the cross-entropy H(p,q) is defined as:

(3) $$H(p,q)=-\sum_{i=1}^{n}p\left(x_{i}\right)\log(q\left(x_{i}\right)),$$

Substituting (1) and (2) to (3), the final cross-entropy loss for $p(x_{i})=1$ can be simplified as

(4) $$H(p,q)=-\log(q\left(x_{i}\right)).$$

**Figure 2:**
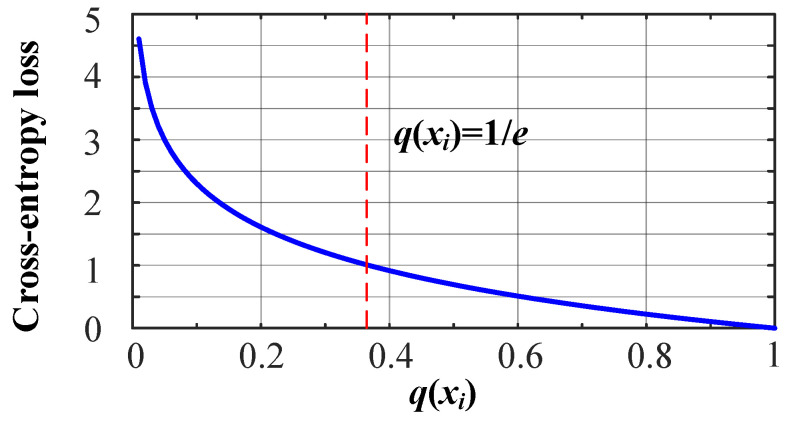
Simplified cross-entropy loss.

Figure 2 shows the curve of simplified cross-entropy loss. We can see that $q<\frac{1}{e}$ when H(p,q)>1. Moreover, the larger the *q*, the smaller the cross-entropy H(p,q) is. It indicates that the difference between the true value and the predicted value will be smaller with more accurate classification.

### 2.3. Attention Mechanism

**Figure 3:**
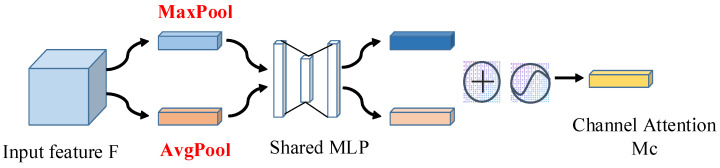
Channel attention module.

In recent years, a series of attention mechanisms have been proposed to improve the performance of the deep learning models, such as channel attention mechanism (CAM) [26], spatial attention mechanism (SAM) [3], mixed attention [27,28], etc. The attention mechanism focuses on the important part of the obtained information. For example, the channel attention mechanism focuses on the correlation between different channels. It captures and reinforces the important features on each channel. The spatial attention mechanism focuses on the areas associated with the task. More succinctly, channel attention mechanisms focus on what is important and spatial attention mechanisms focus on where is important. It is found in [29] that channel-related information is more important than spatial information when embedding the attention module of point cloud features. Therefore, in this paper, we mainly improve the model performance by adding the channel attention mechanism—see Figure 3.

## 3. Proposed Method

In this section, we improve the ROPNet by introducing the revised cross-entropy and the channel attention mechanism.

### 3.1. Modification of Loss Function

In most overlap-based point cloud registration models including ROPNet, the overlap loss is defined as:

(5) $$\begin{array}{cc} loss_{0} & =H_{1}(p_{1},q_{1})+H_{2}(p_{2},q_{2})\\ \; & =\frac{1}{2N}\sum_{i}\sum_{j}p_{1}\cdot \log\left(q_{1}\right)+\frac{1}{2M}\sum_{i}\sum_{j}p_{2}\cdot \log\left(q_{2}\right), \end{array}$$

where $H_{1}\left(p_{1},q_{1}\right)$, $H_{2}\left(p_{2},q_{2}\right)$ are the overlap supervisory losses of the two point clouds. *N* and *M* are the number of points in the source and target point clouds. $p_{1}$, $p_{2}$ are the true distribution of the source and target point clouds, and $q_{1}$, $q_{2}$ are the predicted probability distribution of the source and target point clouds, respectively.

Next, we modify the loss function to reduce the cross-entropy. Since the overlap between the source and target point clouds is high in most point cloud registration tasks, we mainly consider the case when $\frac{1}{e}\leq q\leq 1$. We can see from Figure 2 that 0≤H(p,q)≤1 when $\frac{1}{e}\leq q\leq 1$. According to the square sum inequality, that is, for any $a,b\in R,{\left(a+b\right)}^{2}\geq 2ab$, we propose a new loss function shown by Definition 1.

**Definition** **1.** *An improved loss function with cross-entropy is defined as:*

(6) $$\begin{array}{cc} loss_{1} & =H_{1}(p_{1},q_{1})\times H_{2}(p_{2},q_{2})\\ \; & =\frac{1}{2N}\sum_{i}\sum_{j}p_{1}\cdot \log\left(q_{1}\right)\times \frac{1}{2M}\sum_{i}\sum_{j}p_{2}\cdot \log\left(q_{2}\right), \end{array}$$

*We prove that the proposed loss function (6) performs better than the original one (5) by comparing the absolute value of the derivative of $loss_{0}$ and $loss_{1}$ as follows.*

Denote $H_{k}\left(p_{k},q_{k}\right)$ as any one of the cross-entropy loss, where k=1,2. According to (4), the cross-entropy loss can be expressed as

(7) $$H_{k}\left(p_{k},q_{k}\right)=-\log\left(q_{k}\left(x_{i}\right)\right).$$

Take the derivative of $H_{k}\left(p_{k},q_{k}\right)$. It can be written as

(8) $$\begin{array}{cc} \frac{\partial H_{k}\left(p_{k},q_{k}\right)}{\partial x_{j}} & =\frac{\partial H_{k}\left(p_{k},q_{k}\right)}{\partial q_{k}\left(x_{i}\right)}\times \frac{\partial q_{k}\left(x_{i}\right)}{\partial x_{i}}=\frac{\partial -\log(q_{k}\left(x_{i}\right))}{\partial q_{k}\left(x_{i}\right)}\times \frac{\partial \frac{e^{{}_{{}_{x_{i}}}}}{\sum_{j=1}^{n}e^{{}_{{}_{x_{j}}}}}}{\partial x_{i}}\\ \; & =-\frac{1}{q_{k}\left(x_{i}\right)}\times \frac{\frac{\partial e^{{}_{{}_{x_{i}}}}}{\partial x_{j}}\times \sum_{j=1}^{n}e^{{}_{{}_{x_{j}}}}-e^{{}_{{}_{x_{i}}}}\times e^{{}_{{}_{x_{j}}}}}{(\partial \sum_{j=1}^{n}e^{{}_{{}_{x_{j}}}}{)}^{2}}\\ \; & =-\frac{\sum_{j=1}^{n}e^{{}_{{}_{x_{j}}}}}{e^{{}_{{}_{x_{i}}}}}\times \frac{\frac{\partial e^{{}_{{}_{x_{i}}}}}{\partial x_{j}}\times \sum_{j=1}^{n}e^{{}_{{}_{x_{j}}}}-e^{{}_{{}_{x_{i}}}}\times e^{{}_{{}_{x_{j}}}}}{(\partial \sum_{j=1}^{n}e^{{}_{{}_{x_{j}}}}{)}^{2}}. \end{array}$$

When i=j, $\partial e^{{}_{{}_{x_{i}}}}/\partial x_{j}=\partial e^{{}_{{}_{x_{j}}}}/\partial x_{j}$. (7) can be written as

(9) $$\begin{array}{cc} \frac{\partial H_{k}\left(p_{k},q_{k}\right)}{\partial x_{j}} & =-(1-\frac{e^{{}_{{}_{x_{j}}}}}{\sum_{j=1}^{n}e^{{}_{{}_{x_{j}}}}})\\ \; & =-(1-q_{k}\left(x_{j}\right))\\ \; & =-1+q_{k}\left(x_{j}\right). \end{array}$$

When i≠j, $\partial e^{{}_{{}_{x_{i}}}}/\partial x_{j}=0$. The derivation of $H_{k}\left(p_{k},q_{k}\right)$ with i≠j can be written as

(10) $$\frac{\partial H_{k}\left(p_{k},q_{k}\right)}{\partial x_{j}}=-\frac{e^{{}_{{}_{x_{j}}}}}{\sum_{j=1}^{n}e^{{}_{{}_{x_{j}}}}}=q_{k}\left(x_{j}\right).$$

Combining (7) and (9), the derivation of the loss function in different conditions can be expressed as

(11) $$\frac{\partial H_{k}\left(p_{k},q_{k}\right)}{\partial x_{j}}=\left\{\begin{array}{cc} q_{k}\left(x_{j}\right) & i\neq j\\ -1+q_{k}\left(x_{j}\right) & \;\mathrm{if}\;i=j \end{array}.\right.$$

Then, to compare the values of the original and proposed loss functions, we compute the derivation of $loss_{0}$ and $loss_{1}$.We denote $y_{0}$ and $y_{1}$ as the derivation of $loss_{0}$ and $loss_{1}$, respectively. Substituting (1) and (11), $y_{0}$ and $y_{1}$ can be calculated as

(12) $$y_{0}=-1+q_{1}\left(x_{j}\right)-1+q_{2}\left(x_{j}\right)$$

(13) $$y_{1}=\left(-1+q_{1}\left(x_{j}\right)\right)H_{2}\left(p_{2},q_{2}\right)+H_{1}\left(p_{1},q_{1}\right)\left(-1+q_{2}\left(x_{j}\right)\right)$$

According to Figure 2, when $\frac{1}{e}\leq q\leq 1$, it has $0\leq H(p,q)=-\log(q\left(x_{i}\right))\leq 1$. Since $0\leq q_{1j},q_{2j}\leq 1$, we have $y_{0}\leq y_{1}\leq 0$. Therefore, $\left|y_{1}\right|\leq \left|y_{0}\right|$.

The above analysis shows that the absolute value of the derivative of our proposed loss function is smaller than that of the original loss function. It means that the proposed loss function has a better performance. Moreover, since $q\geq \frac{1}{e}$ in most point cloud alignment tasks, the proposed loss function can be generalized in more tasks based on overlapping point regions.

### 3.2. Channel Attention-Based ROPNet

**Figure 4:**
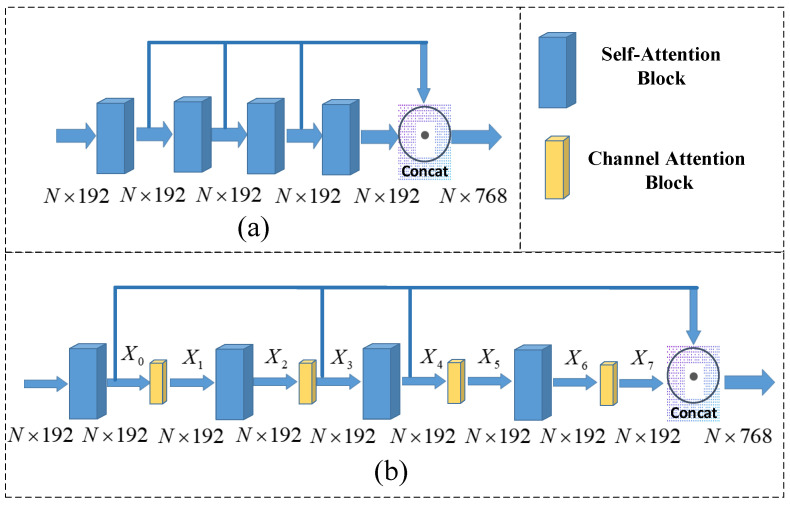
(**a**) Initial TMFR structure; (**b**) improved structure.

Figure 4a shows the original structure of TMFR module, which contains offset-attention blocks. The offset attention block is used as the self-attention to generate global features. The TMFR module fuses the multi-scale features obtained from multiple attention modules. However, the original self-attention module only extracts global features, but ignores the local features. Hence, to make the features more complete, we add a channel attention module after each of the original attention module. As a matter of fact, there are a lot of ways to add the channel attention model. Empirically, we use a hybrid attention mechanism as shown in Figure 4b. The improvement of the proposed model with the attention mechanism can be demonstrated by experiments presented in Section 4.

### 3.3. Overall Improved Architecture

**Figure 5:**
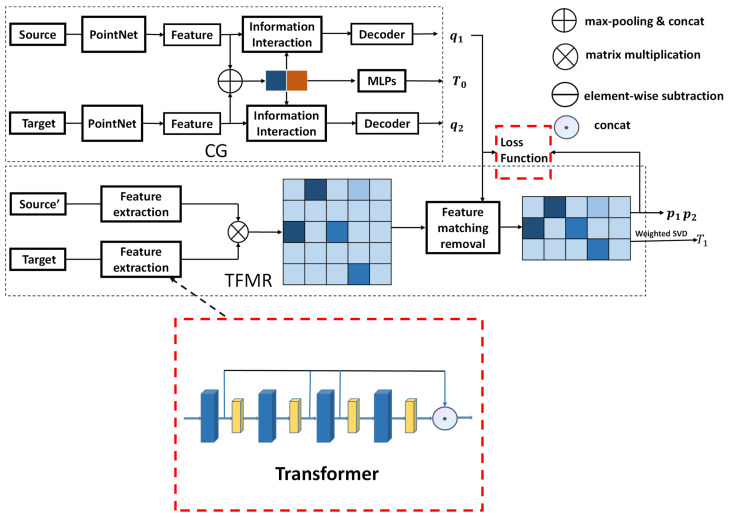
An overall architecture of the improved ROPNet.

An overall architecture of the improved ROPNet is presented in Figure 5. Source’ represents the point cloud after the initial transformation of the source point cloud. $T_{0}$ is the initial transformation matrix, $T_{1}$ is the final transformation matrix. The improvement has two aspects. One is that we added a channel attention mechanism in the feature extraction part to focus more on the local features. The other is that an improved loss function presented in Section 3.2 is used after obtaining the true distribution $p_{1}$ and $p_{2}$.

## 4. Experiments

We conducted a series of experiments to demonstrate the effectiveness and advantage of our proposed method. Firstly, we introduce the dataset and the evaluation metrics. Secondly, we compared our proposed loss function with the original one by experiments. Thirdly, we conducted the ablation experiment to show the effectiveness of our proposed model with attention mechanism. Finally, we use both the improved model and the improved loss function to show the advantage of our proposed method. We keep the same experimental settings, parameters, and evaluation indicators with [15].

### 4.1. Dataset and Evaluation Metrics

As with [15], this paper used dataset ModelNet40 [30], including 40 categories and 12311 CAD models. We trained the model in the first 20 categories and tested in the remaining 20. Since there are some symmetrical categories in ModelNet40, we divided the data into two categories. One is the total objects (TO), and the other is the asymmetric objects (AO). No symmetrical objects, such as tent or vase, exist in AO. According to RPMNet [15], we generated the source and target point clouds by sampling twice to take 1024 points. We randomly generated three Euler angles within $\left[0^{\circ },45^{\circ }\right]$ and translations within [−0.5, 0.5] on each axis. Then, we projected all points to a random direction and deleted thirty percent of them to generate a partial point cloud. In addition, the model is trained on noisy data. The noise is sampled from $N\left(0,0.01^{2}\right)$ and clipped to [−0.05, 0.05].

In the following experiments, we set the average isotropic rotation and translation error as the evaluation metrics, which are

(14) $$Error\left(R\right)=arccos\frac{tr({\hat{R}}^{-1}R)}{2}$$

(15) $$Error\left(t\right)={∥{\hat{R}}^{-1}t-\hat{t}∥}_{1}.$$

$\hat{R}$ and $\hat{t}$ are the ground truth transformation, while *R* and *t* are the predicted transformation. The mean absolute error MAE(R) is used for the rotation matrix and MAE(t) is used for the translation vector.

### 4.2. Improvement by New Loss Function

In this subsection, we compare the results obtained from different loss functions. In the following tables and legends of the figures, denote loss*loss as the proposed loss function, and loss + loss as the original loss function. Denote 0*loss as the loss functions without considering the overlapping region.

**Figure 6:**
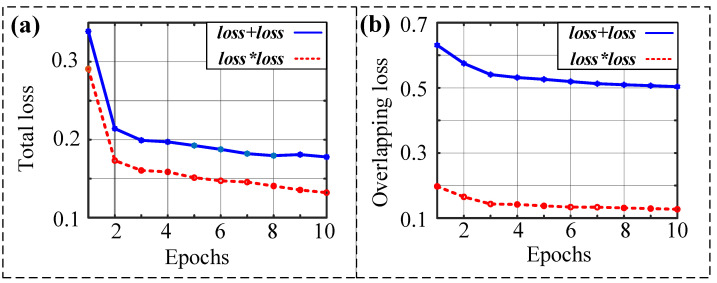
Comparison of loss function decline in the case of 10 epochs: (**a**) total loss; (**b**) overlapping loss.

First, let us observe the cross-entropy loss convergence of different loss functions under 10 epochs. Figure 6a depicts the total loss function of the two methods. We can see that the total loss of the proposed loss function is smaller than the total loss of the original one. In addition, the starting point of the decline of the proposed loss function is lower than the original starting point. Figure 6b shows the overlapping loss calculated by different loss functions. It can be seen that both curves decrease with the increase in epochs. The trend of the two curves is consistent. More importantly, the product of cross-entropy loss, i.e., $loos_{1}$, is smaller than the sum of cross-entropy loss functions, i.e., $loss_{0}$. The above results illustrate that the proposed loss function has a better performance.

**Figure 7:**
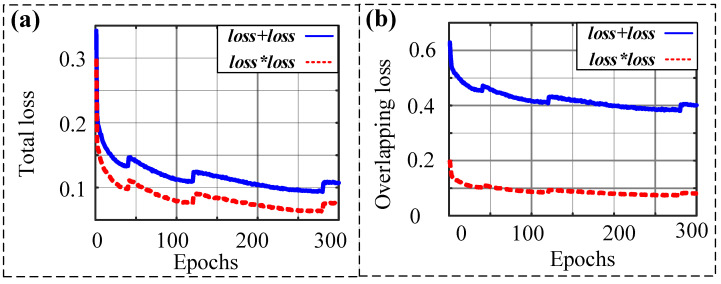
Comparison of loss function decline in the case of 300 epochs: (**a**) total loss; (**b**) overlapping loss.

In order to obtain more reliable results, we calculate different loss functions after training for 300 epochs. The convergence of the total loss and the overlapping loss are shown in Figure 7a,b. The results show that the two loss functions have the same trend and the proposed loss function is smoother and smaller than the original one.

**Figure 8:**
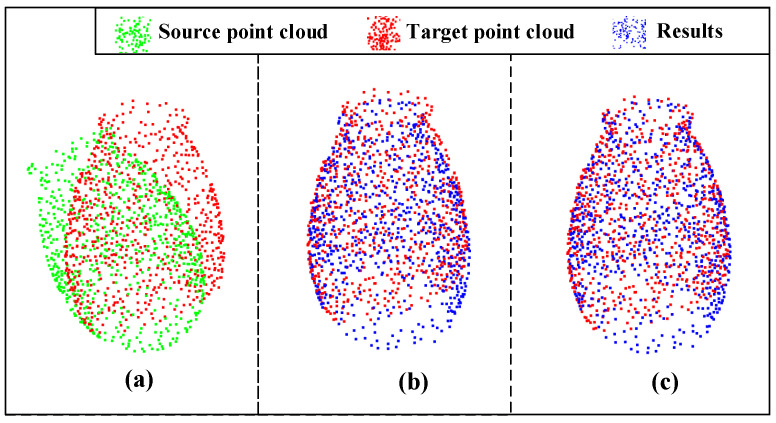
Registration visualization under 300 epochs: (**a**) input; (**b**) ROPNet; (**c**) ours.

**Figure 9:**
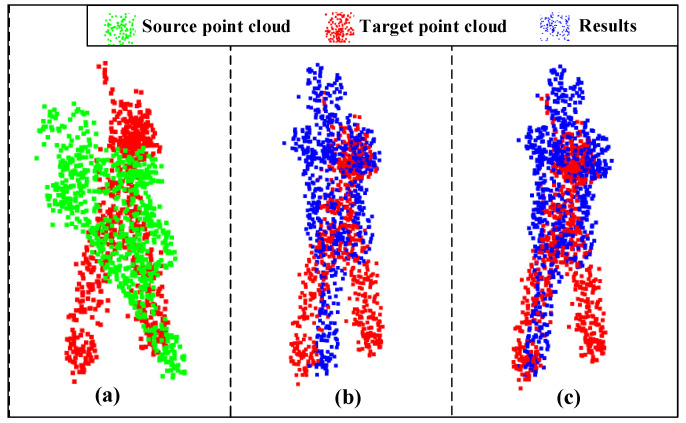
Registration visualization under 300 epochs with Gaussian noise: (**a**) input; (**b**) ROPNet; (**c**) ours.

Next, we conducted two experiments to show the evaluation metrics of different loss functions. Table 1 shows the results of the evaluation metrics in the original and proposed loss functions after training for 300 epochs, and Table 2 shows the results of the evaluation metrics after training for 600 epochs. It is important to note that our table is divided into two parts: the top half being the results obtained in the noiseless condition and the bottom half in the noisy condition. We can see that in both tables, most of the metrics of the proposed loss function are smaller than the metrics of the original loss function, which indicates that our proposed loss function performs better than the original one. Furthermore, we found that in most cases, the larger the original value of the metrics, the better error reduction is. It is also worth noticing that comparing with the loss function without considering the overlapping areas, the proposed loss function improves the accuracy of the registration significantly. Taking the results training for 300 epochs as an example, the analysis results can be tested via alignment visualization results for some data, as shown in Figure 8 and Figure 9. From these figures, we can see that our proposed method improves the alignment effects.

### 4.3. Ablation Experiment

In this subsection, we compare the results of the original ROPNet model and the ROPNet model with channel attention mechanism. Then, we analyze the results obtained from our proposed solution.

Table 3 shows the results of the evaluation metrics in the original and proposed ROPNet model. Compared with the original ROPNet, the results show that most of the metrics, especially the metrics in Schemes 1 and 3 are smaller than the original one. The larger the original value of metrics, the better the performance of the proposed model.

**Figure 10:**
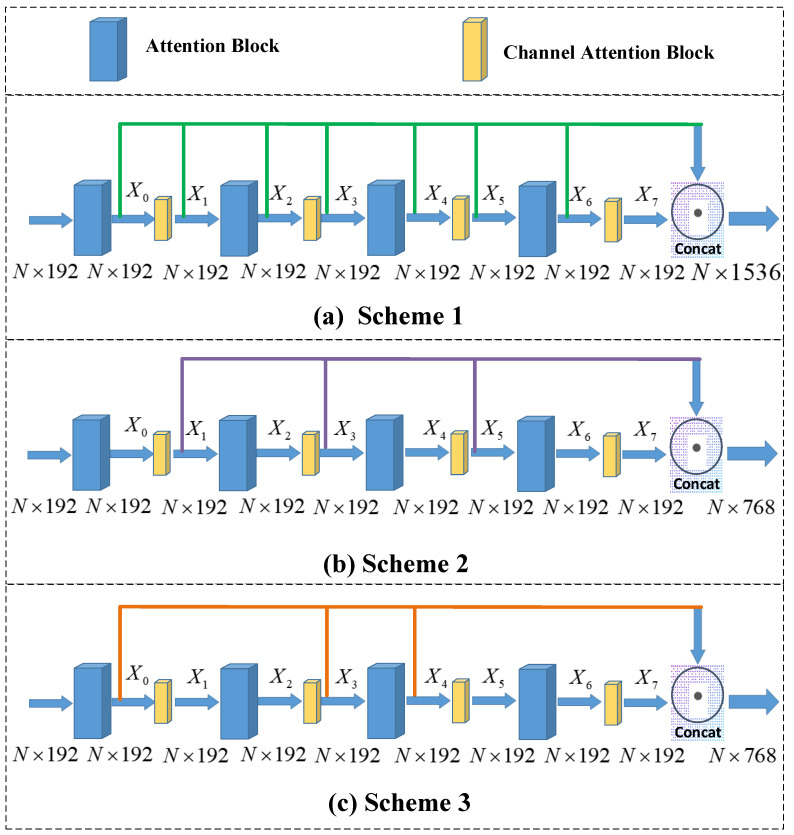
Comparison of different schemes: (**a**) Scheme 1 concat[x0, x1, x2, x3, x4, x5, x6, x7]; (**b**) Scheme 2 concat[x1, x3, x5, x7]; (**c**) Scheme 3 concat[x0, x3, x4, x7].

Next, we discuss the results when selecting different positions to fuse the features. As mentioned in Section 3.2, there are a lot of ways to add the channel attention model. Figure 10 illustrates three fusion schemes with different positions. In specific, Figure 10a shows the stepwise fusion method, Figure 10b shows the feature extracted through the channel attention module, and Figure 10c shows a mixed method. Table 3 depicts the results of feature fusion with different positions. It can be seen that Scheme 1 and Scheme 3 has better performance than Scheme 2, because Scheme 2 ignores the global information. Moreover, Scheme 1 fuses multiple layers of feature and leads to the feature redundancy. So, we choose Scheme 3 as the feature fusion method.

### 4.4. Combination of Two Improvements

In this subsection, we combine the proposed loss function with the improved model to show the effectiveness of our proposed method on ModelNet40.

Table 4 shows the results of evaluation metrics in different methods. We can see that the results with the improved loss function and improved model has the best performance comparing with others. When comparing the metric results in AO utilizing both the improved loss function and model with the original metric results, we can see from Table 4 that Error(R) and Error(t) with no noise are reduced by 4.83% and 14.16%. Error(R) and Error(t) with noise are reduced by 9.60% and 8.89%. When comparing the metric results utilizing both the improved loss function and model with the results only utilizing the improved loss function, Error(R) and Error(t) with no noise are reduced by 4.10% and 11.82%. The Error(R) and Error(t) with noise are reduced by 5.34% and 9.56%. When comparing the metric results utilizing both the improved loss function and model with the results only utilizing the improved model, Error(R) and Error(t) with no noise are reduced by 7.20% and 20.18%. The Error(R) and Error(t) with noise are reduced by 5.69% and 9.56%. The results show that the proposed method with the improved loss function and the improved model is effective in improving the performance of the model.

**Figure 11:**
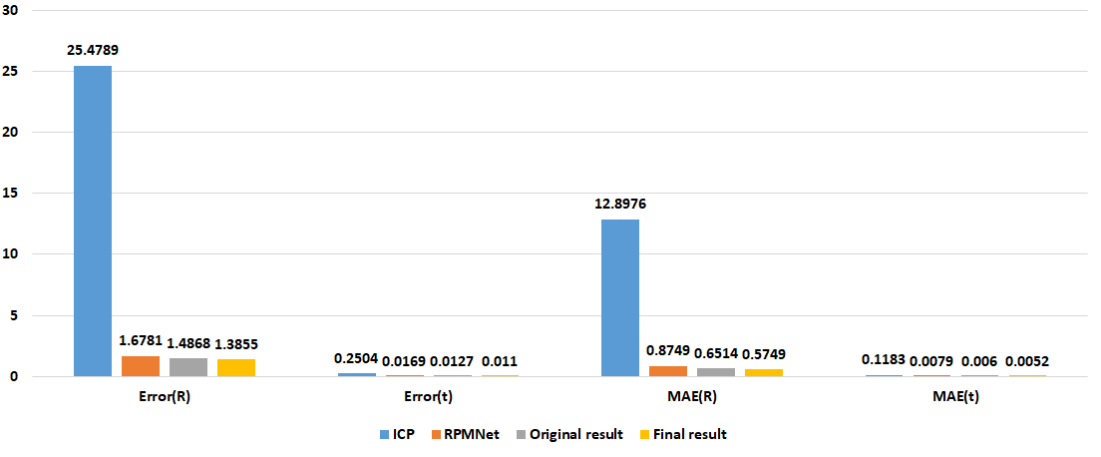
Registration results on ModelNet40.

**Figure 12:**
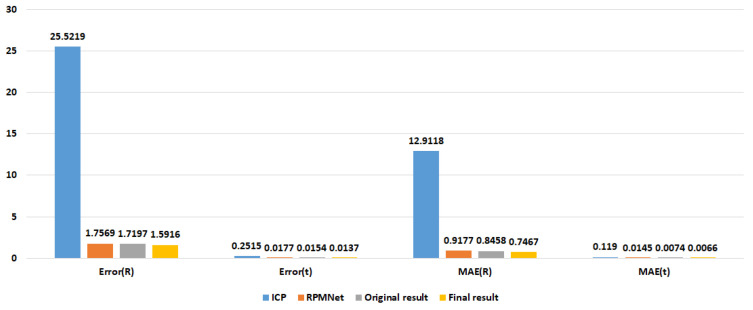
Registration results on ModelNet40 with Gaussian noise.

Figure 11 and Figure 12 show the comparison results of ICP, RPMNet, ROPNet, and the proposed model in the form of bar charts. We can see that our method is superior to both RPMNet and the original ROPNet. When comparing the metric results with RPMNet, Error(R) and Error(t) with no noise are reduced by 17.44% and 34.91%. The Error(R) and Error(t) with noise are reduced by 9.41% and 22.60%, respectively. Comparing with ICP, our method performs better, the Error(R) and Error(t) is reduced by a factor of more than ten.

**Figure 13:**
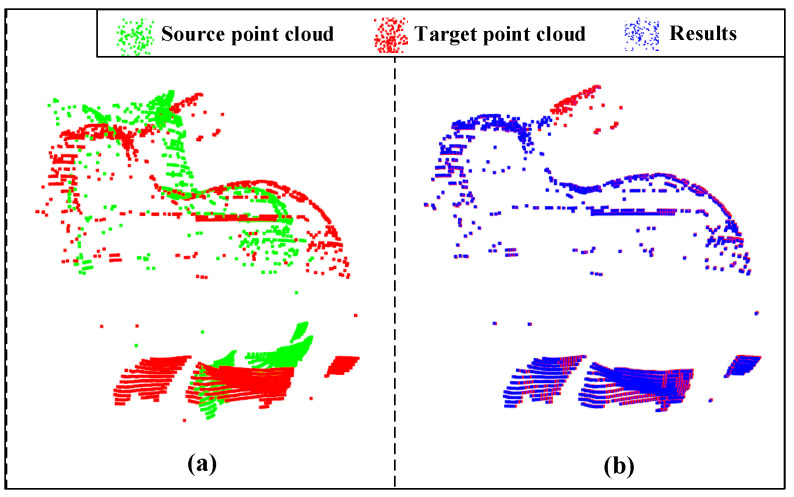
Registration results of Stanford bunny model: (**a**) input; (**b**) result error (R): 0.3403; Error(t): 0.0219.

We also tested our method on the Stanford Bunny dataset. The Stanford Bunny dataset is composed of 35,947 points. We rotated the Bunny model by 30 degrees around the y-axis. Then, 1500 points are selected from the original point cloud and the point cloud after rotation as the source and target point clouds. The final result is shown in Figure 13.

## 5. Conclusions

In this paper, we designed a new way of using the cross-entropy loss, which integrates two cross-entropy losses by the product instead of sum. To verify the effectiveness of this loss, we applied it to the point cloud registration network ROPNet. In addition, we improve the original ROPNet model by introducing the channel attention mechanism, which focus on the local features. A large number of experiments show that the proposed method improves the accuracy and efficiency of the point cloud registration task. Compared to the original ROPNet, the Error(R) and Error(t) with noise are reduced by 4.83% and 14.16%. Compared to the RPMNet, the Error(R) and Error(t) with noise are reduced by 9.41% and 22.60%. Compared to other traditional methods such as ICP the Error(R) and Error(t) are even reduced by more than ten times. In this way, the proposed method is expected to contribute to more point cloud registration models.

## Figures and Tables

**Table 1:** Results of metrics with different loss functions training for 300 epochs.

	AO				TO			
	**Error(R)**	**Error(t)**	**MAE(R)**	**MAE(t)**	**Error(R)**	**Error(t)**	**MAE(R)**	**MAE(t)**
noiseless								
loss*loss	**1.2994**	**0.0138**	**0.6186**	**0.0066**	**1.6687**	**0.0146**	**0.7634**	**0.0069**
loss+loss	1.3926	0.0141	0.6287	0.0067	1.7064	0.0151	0.7951	0.0072
noise								
loss*loss	**1.47**	**0.016**	**0.7378**	**0.0076**	**1.8856**	**0.0178**	**0.9303**	**0.0085**
loss+loss	1.5239	0.0163	0.7516	0.0078	1.9385	0.0179	0.9606	0.0086

**Table 2:** Results of metrics with different loss functions training for 600 epochs.

	AO				TO			
	**Error(R)**	**Error(t)**	**MAE(R)**	**MAE(t)**	**Error(R)**	**Error(t)**	**MAE(R)**	**MAE(t)**
noiseless								
loss+loss	1.191	0.0113	0.5089	0.0053	1.4868	0.0127	0.6514	0.006
0*loss	2.8917	0.0311	1.5149	0.0148	3.2228	0.0327	1.6547	0.0156
loss*loss	**1.1819**	**0.011**	**0.5001**	**0.0052**	**1.4255**	**0.0122**	**0.6241**	**0.0058**
noise								
loss+loss	1.3935	**0.0135**	0.6502	**0.0065**	1.7197	0.0154	0.8458	0.0074
0*loss	3.0078	0.0327	1.6225	0.0156	3.5492	0.0362	1.8584	0.0173
loss*loss	**1.3308**	0.0136	**0.6305**	0.0066	**1.6164**	**0.0145**	**0.7754**	**0.0070**

**Table 3:** Results of metrics with different models.

	AO				TO			
	**Error(R)**	**Error(t)**	**MAE(R)**	**MAE(t)**	**Error(R)**	**Error(t)**	**MAE(R)**	**MAE(t)**
noiseless								
Original result	1.191	0.0113	0.5089	0.0053	1.4868	0.0127	0.6514	0.006
Scheme 1	**1.1754**	**0.0101**	**0.4759**	**0.0047**	1.4637	0.0121	0.6508	0.0057
Scheme 2	1.233	0.0113	0.5304	0.0054	1.4461	0.0123	0.6536	0.0058
Scheme 3	1.2215	0.0114	0.5057	0.0053	**1.3976**	**0.0117**	**0.6215**	**0.0056**
noise								
Original result	1.3935	0.0135	0.6502	0.0065	1.7197	0.0154	0.8458	0.0074
Scheme 1	**1.2797**	**0.0128**	**0.6055**	**0.0062**	1.7157	0.0149	0.8235	0.0072
Scheme 2	1.3741	0.0133	0.6389	0.0065	1.6765	0.0152	0.8302	0.0073
Scheme 3	1.3357	0.0136	0.6162	0.0066	**1.5287**	**0.0141**	**0.7399**	**0.0068**

**Table 4:** Overall improved results on ModelNet40

	AO				TO			
	**Error(R)**	**Error(t)**	**MAE(R)**	**MAE(t)**	**Error(R)**	**Error(t)**	**MAE(R)**	**MAE(t)**
noiseless								
Original result	1.191	0.0113	0.5089	0.0053	1.4868	0.0127	0.6514	0.006
loss*loss	1.1819	0.011	0.5001	0.0052	1.4255	0.0122	0.6241	0.0058
Scheme 3	1.2215	0.0114	0.5057	0.0053	1.3976	0.0117	0.6215	0.0056
Final result	**1.1335**	**0.0097**	**0.4717**	**0.0046**	**1.3855**	**0.011**	**0.5749**	**0.0052**
noise								
Original result	1.3935	0.0135	0.6502	0.0065	1.7197	0.0154	0.8458	0.0074
loss*loss	1.3308	0.0136	0.6305	0.0066	1.6164	0.0145	0.7754	0.0070
Scheme 3	1.3357	0.0136	0.6162	0.0066	**1.5287**	0.0141	**0.7399**	0.0068
Final result	**1.2597**	**0.0123**	**0.5953**	**0.0059**	1.5916	**0.0137**	0.7467	**0.0066**

## Data Availability

Not applicable
